# Differential role of GPR142 in tryptophan-mediated enhancement of insulin secretion in obese and lean mice

**DOI:** 10.1371/journal.pone.0198762

**Published:** 2018-06-11

**Authors:** Yoko Ueda, Hiroshi Iwakura, Mika Bando, Asako Doi, Hiroyuki Ariyasu, Hidefumi Inaba, Shuhei Morita, Takashi Akamizu

**Affiliations:** The First Department of Medicine, Wakayama Medical University, Wakayama, Japan; Consiglio Nazionale delle Ricerche, ITALY

## Abstract

Tryptophan is reportedly the most potent agonist for GPR142. Glucose-stimulated insulin secretion (GSIS) from pancreatic beta cells are enhanced by GPR142-mediated signal. It is not clear, however, if GPR142-mediated signals is solely attributable to GSIS enhancement after tryptophan load in various pathophysiological settings. This study aims to reveal the significance of GPR142 signaling in tryptophan-mediated GSIS enhancement in normal and obese mice. Tryptophan significantly improved glucose tolerance in both lean and DIO mice, but the extent of improvement was bigger in DIO mice with augmented glucose-stimulated insulin secretion (GSIS) enhancement. The same results were obtained in ob/ob mice. GPR142 deletion almost completely blocked tryptophan actions in lean mice, suggesting that GPR142 signaling was solely responsible for the GSIS enhancement. In obese GPR142KO mice, however, a significant amount of tryptophan effects were still observed. Calcium-sensing receptors (CaSR) are also known to recognize tryptophan as ligand. Expression levels of CaSR were significantly elevated in the pancreas of DIO mice, and CaSR antagonist further blocked tryptophan’s actions in DIO mice with GPR142 deletion. Although GPR142 signaling had a major role in tryptophan recognition for the enhancement of GSIS in lean mice, other pathways including CaSR signaling also had a significant role in obese mice, which seemed to contribute to the augmented enhancement of GSIS by tryptophan in these animals.

## Introduction

The emerging role of nutrient-sensing receptors in glucose metabolisms and body energy homeostasis has gained the attention of many researchers [1]. Endocrine cells in the gastrointestinal tract sense the nutrients by the nutrient-sensing receptors, releasing various peptide hormones, including ghrelin, cholecystokinin, peptide YY, glucagon-like peptide-1, and gastric inhibitory peptide, to regulate food intake or insulin secretion. GPR120, a long-chain fatty acid receptor, is expressed in the gastric X/A-like cells and intestinal L cells to regulate the secretion of both ghrelin [2, 3] and GLP-1 [4]. Some nutrient-sensing receptors, such as long-chain fatty acid receptor GPR40, are also expressed in pancreatic beta cells controlling insulin secretion [5], and are considered to be potential drug-targets for treatment of diabetes [6].

We previously found that L-tryptophan (L-Try) strongly stimulates ghrelin secretion from both the ghrelin-producing cell line, MGN3-1 [7] and primary cultured stomach epithelial cells [8]. Comprehensive profiling of G-protein coupled receptors (GPCRs) in MGN3-1 revealed that recently deorphanaized aromatic amino acid receptor, GPR142 [9] is highly expressed in the cells [8]. Knockdown of GPR142 by siRNA significantly attenuated L-Try-induced ghrelin secretion, suggesting that GPR142 plays a key role in the L-Try-mediated ghrelin secretion [8].

GPR142 was originally reported as a novel rhodopsin family G protein-coupled receptors (GPCRs) by genome database search [10]. Susens et al. reported that GPR142 was expressed in mouse testis, brain, spleen, liver, and kidney by Northern blot analysis [11]. Meanwhile, Lin et al. reported that GPR142 was expressed at the highest levels in the pancreatic islets, followed by in the stomach, the duodenum, the ileum, and the jejunum [12]. They showed that tryptophan significantly enhanced glucose-stimulated insulin secretion (GSIS) in vivo and from isolated islets and improved glucose tolerance in mice. The enhancement was via direct effects on the beta cells, and partly via stimulation of gastric inhibitory peptide (GIP) secretion. This was not observed in GPR142 knockout (KO) mice [12].

Besides GPR142, calcium-sensing receptor (CaSR) can recognize aromatic amino acids including phenylalanine and tryptophan [13–17]. It was originally cloned from bovine parathyroid gland, and senses extracellular Ca^2+^ levels to regulate parathyroid hormone secretion [18]. CaSR is also expressed in the gastrointestinal tract [19–21] and pancreatic beta cells [22, 23] to regulate gastric acid [24] and gastrointestinal hormones including CCK, GIP, GLP-1, and PYY by sensing aromatic amino acids levels [14, 25–27].

It is not clear, however, if GPR142-mediated signals is solely attributable to GSIS enhancement after tryptophan load in various pathological settings. This study aims to reveal the significance of GPR142 signaling in tryptophan-mediated GSIS enhancement in obese mice.

## Materials and methods

### Animals

We used male C57/BL6 mice (Japan SLC, Inc., Shizuoka, Japan). Animals were maintained on a 12-h light/12-h dark cycle and fed either a standard diet (SD; CE-2, 352 kcal/100g; Japan CLEA, Tokyo, Japan) or an HFD containing 60% fat/kcal (Research Diet Inc., New Brunswick, NJ) as indicated. Ob/ob mice and their control littermates were purchased from Japan SLC, Inc. GPR142KO mice (C57BL/6 background) were obtained from Jackson Laboratory (stock no. 026065, Bar Harbor, ME). All experimental procedures were approved by the Wakayama Medical University Committee on Animal Research.

#### Glucose tolerance tests

For glucose tolerance testing, *ad libitum*-fed mice were gavaged with 1.0 g/kg glucose with or without L-tryptophan (150 mM, 0.5 g/kg, dissolved in 15% 2-Hydroxypropyl-β-cyclodextrin), with or without 1μM NPS2143 (Cayman Chemical, MI USA). Blood was sampled from the tail veins before and 30, 60, 90, and 120 min after the injection. Blood glucose levels were determined by the glucose oxidase method using a Glutest sensor (Sanwa Kagaku, Kyoto, Japan). For incretin measurements, blood was obtained from retro orbital veins, and Na_2_EDTA (1mg/ml), aprotinin (1000 KIU/ml), and DPPIV inhibitor (20μl/mL plasma), were added to the samples. Collection of plasma samples for the measurement of ghrelin was performed as reported previously [28].

### Hormone measurements

Insulin concentrations were measured by Ultra Sensitive Mouse Insulin kit (Morinaga, Yokohama, Japan). GIP concentrations were measured by a mouse active GIP ELISA kit (Wako, Osaka, Japan). GLP-1 concentrations were measured by a GLP-1 (7–36) active ELISA kit (Linco Research Inc. St. Charles, MO). Plasma ghrelin concentration was determined by AIA-600II (Tosoh, Tokyo, Japan) as previously described [29].

### Real-time quantitative RT-PCR

Real-time quantitative RT-PCR was conducted as reported [30]. Total RNA was extracted using an RNeasy mini kit (QIAGEN, Hilden, Germany). Reverse transcription (RT) was performed using a high-capacity cDNA reverse transcription kit (Applied Biosystems, Foster City, CA). Real-time quantitative PCR was performed on an ABI PRISM 7500 Sequence Detection System (Applied Biosystems) using the following primers: mouse GPR142, sense, 5’- TGCTGCCTACAGTCAATGGT -3’, antisense, 5’- TGACGATATCTGAAGCCGTG -3’; mouse CaSR, sense, 5’- TCCATTTTGGAGTAGCAGCC -3’, antisense, 5’- GCAGTTGCAGAACTCATCCA -3’ with Power SybrGreen. Data were normalized to the 18 S rRNA content in each sample. Data were presented as relative value.

### Statistical analyses

All values were expressed as the mean ± S.E. Statistical significance of differences in mean values was assessed by Student’s *t*-test. Differences of *p* < 0.05 were considered significant. Statistical analyses were performed using Statcel4 (OMS, Saitama, Japan).

## Results

**Fig 1:**
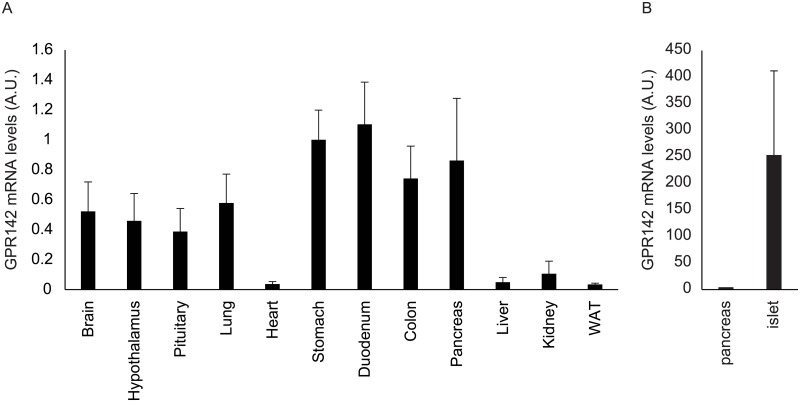
Expression levels of GPR 142 mRNA in the various mouse tissues. GPR142 expression levels determined by quantitative RT-PCR in (A) the various tissues and isolated pancreatic islets (B) of 8-week old wild type C57BL / 6 male mice. n = 6, A.U.: Arbitrary Units.

We first examined GPR142 mRNA levels in various mouse tissues (Fig 1A). Expression levels were high in the stomach, the duodenum, and the pancreas. Substantial levels of expression were also observed in the brain, including the hypothalamus and pituitary, and in the lung (Fig 1A). The GPR142 expression levels in the pancreatic islet were higher than in the pancreas (Fig 1B), suggesting that GPR142 were expressed in pancreatic endocrine cells.

**Fig 2:**
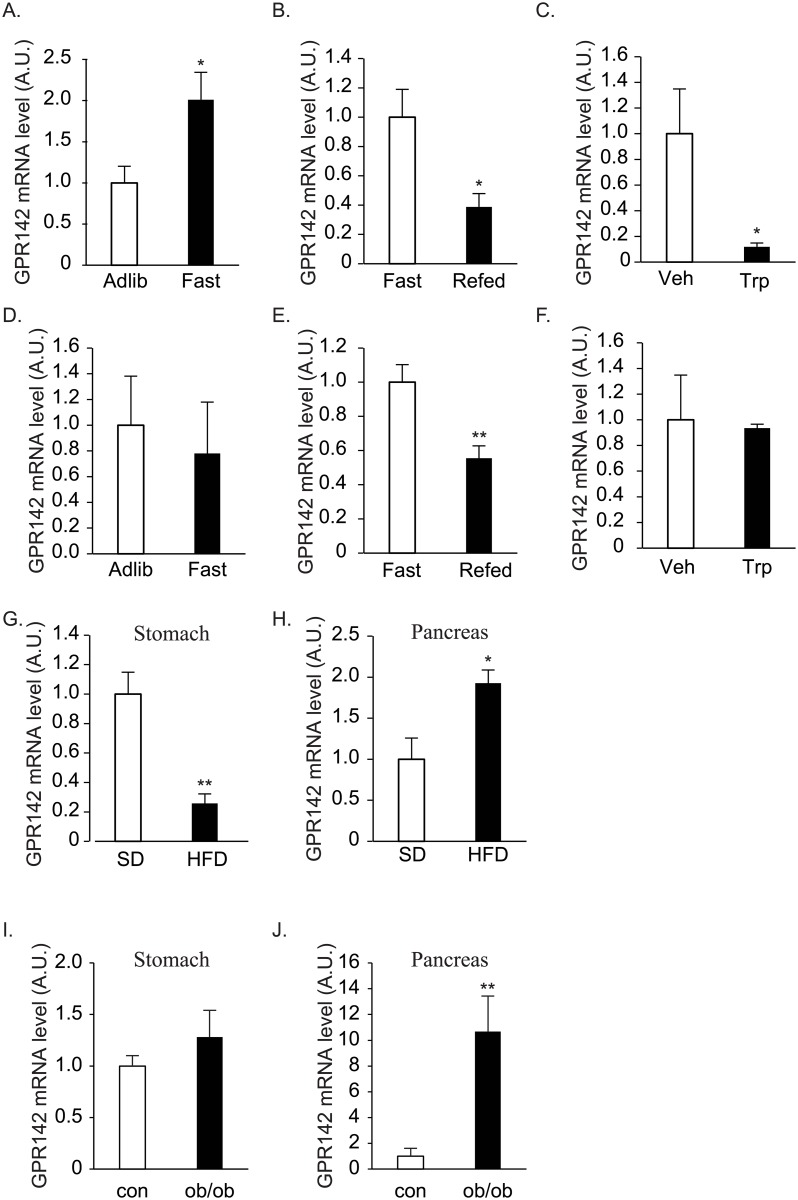
Effects of feeding status and body weight on expression levels of GPR142 mRNA. A-F. The effects of overnight fasting (A, and D) and 2-hours re-feeding (B, and E) on GPR142 expression levels in the stomach (A, and B) and pancreas (D, and E) of nine-week old C57BL / 6 male mice. n = 6, *: p<0.05. C, and F. The effects of tryptophan gavage on GPR142 expression levels in the stomach (C) and pancreas (F). n = 7, *: p<0.05. G, H. GPR142 expression levels in the stomach (G) and pancreas (H) of mice fed with standard diet (SD) and 60% high fat diet (HFD) for eighteen weeks. n = 6, **: p<0.01, *: p<0.05. I, J. GPR142 expression levels in the stomach (I) and pancreas (J) of ad libitum-fed 11-week-old ob/ob and their control (con) littermates. n = 9, **: p<0.01, *: p<0.05.

Next, we examined if nutrient ingestion affects GPR142 expression levels in the stomach and pancreas. In the stomach, GPR142 mRNA expression levels were elevated by fasting (Fig 2A) and suppressed by re-feeding (Fig 2B). Tryptophan ingestion significantly suppressed GPR142 mRNA expression levels in the stomach (Fig 2C), suggesting that the suppression by re-feeding was at least in some part mediated by tryptophan included in the chow.

GPR142 mRNA expression levels in the pancreas were not elevated by fasting (Fig 2D), but were suppressed by re-feeding (Fig 2E). Tryptophan ingestion did not affect GPR142 mRNA levels in the pancreas (Fig 2F).

**Fig 3:**
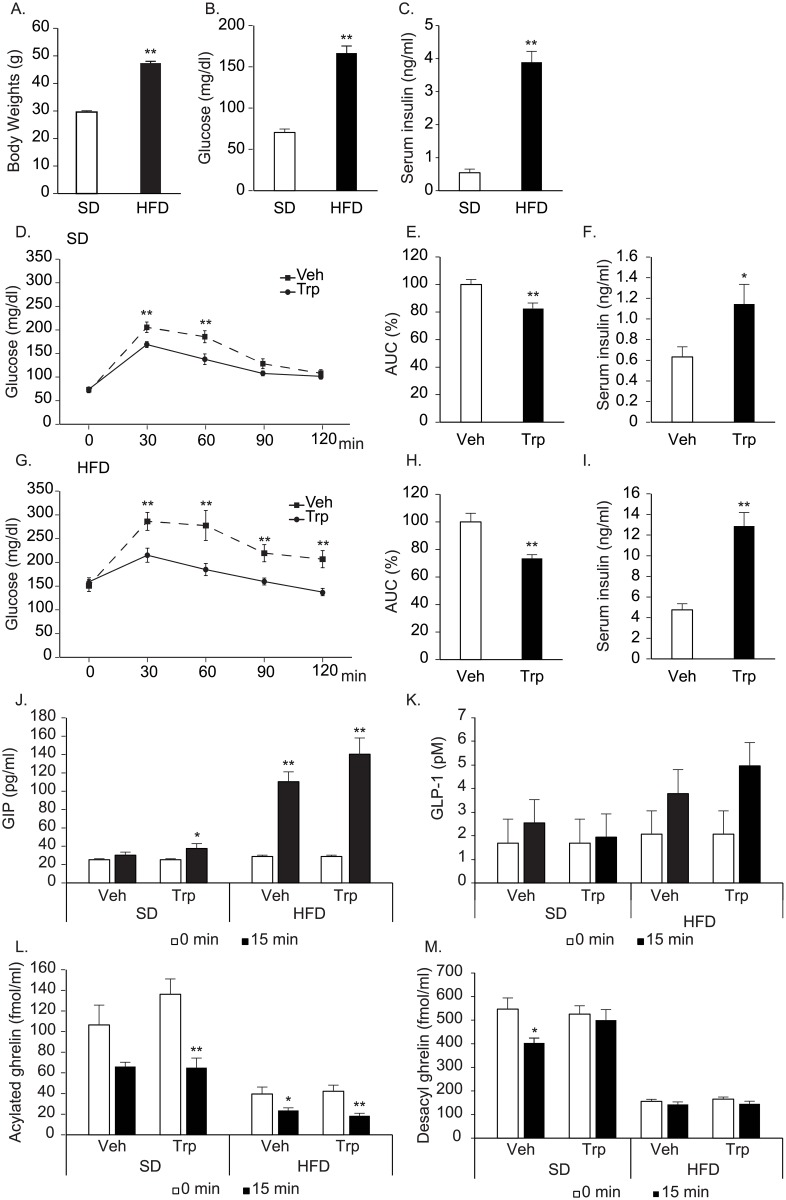
Effects of tryptophan on glucose tolerance in DIO mice. A-C. Body weights (A), basal blood glucose (B), and serum insulin (C) levels in the mice fed with standard diet (SD) and 60% high fat diet (HFD) for eighteen weeks. D, G. Blood glucose levels after oral glucose load (1.0 g/kg) with tryptophan (150mM, 0.5g/kg, Trp) or vehicle (Veh) in 18-week-old mice fed with standard diet (SD, D) or high fat diet (HFD, G). E, H. Area under the curve (AUC) of glucose levels in D and G. F, I, J-M. Serum insulin (F, I), plasma GIP (J), GLP-1(K), acylated ghrelin (L), and desacyl ghrelin (M) concentrations 15 minutes after glucose load in the mice fed with standard diet (SD) or high fat diet (HFD). n = 6–7 per group, **: p<0.01, *: p<0.05.

**Fig 4:**
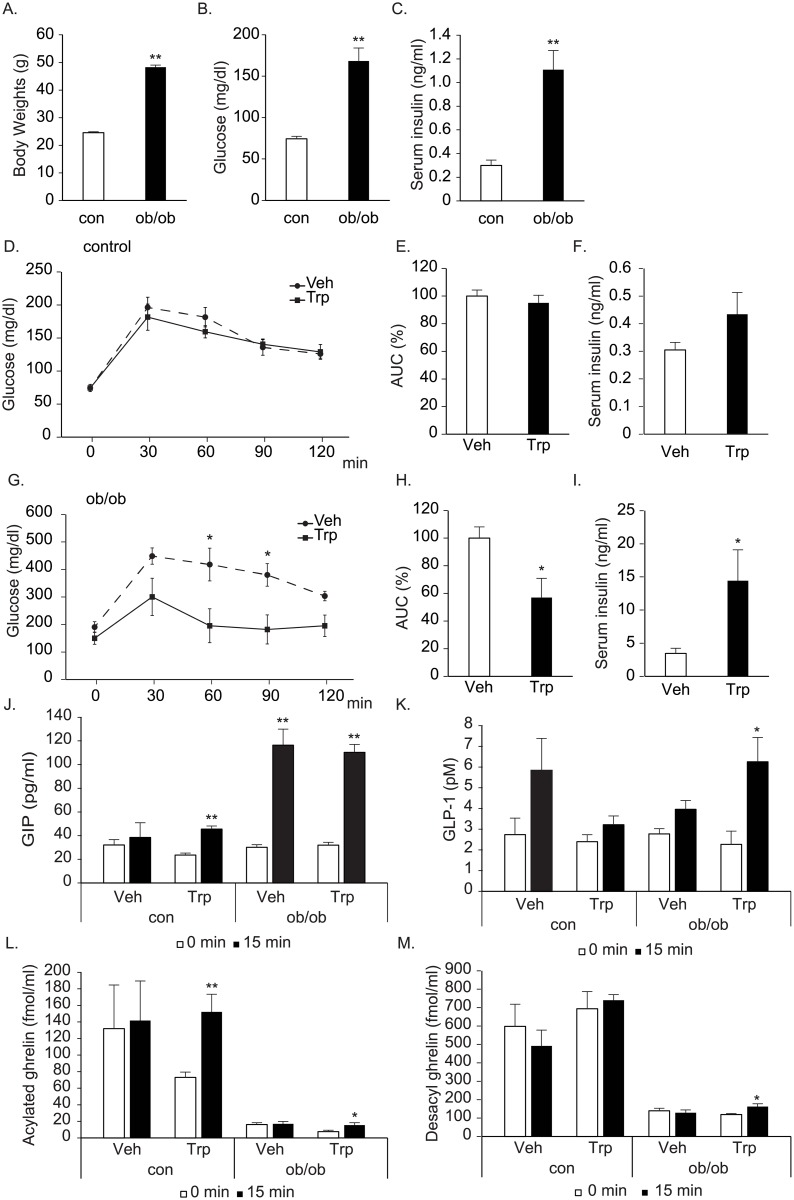
Effects of tryptophan on glucose tolerance in ob/ob mice. A-C. Body weights (A), basal blood glucose (B), and serum insulin (C) levels in 11-weeks old male ob/ob mice and their control littermates. D, G. Blood glucose levels after oral glucose load (1.0 g/kg) with tryptophan (150mM, 0.5g/kg, Trp) or vehicle (Veh) in ob/ob (E) or control mice (G). E, H. Area under the curve (AUC) of glucose levels in D and G. F, I, J-M. Serum insulin (F, I), plasma GIP (J), GLP-1(K), acylated ghrelin (L), and desacyl ghrelin (M) concentrations 15 minutes after glucose load in ob/ob and control (con) mice. n = 6–7 per group, **: p<0.01, *: p<0.05.

We examined GPR142 mRNA expression levels in the stomach and pancreas of diet-induced obesity (DIO) and ob/ob mice to explore if chronic energy balance affects GPR 142 mRNA levels. Body weight of the mice fed with 60% HFD for 14 weeks and ob/ob mice was significantly higher than those of controls (Figs 3A and 4A). In the stomach, the expression levels were significantly lower in the DIO mice (Fig 2H), but not in ob/ob mice (Fig 2H and 2K). The levels were significantly higher in the pancreas of both DIO and ob/ob mice (Fig 2I and 2L).

Previous reports suggested that activation of GPR142 by tryptophan improves glucose tolerance in mice. We examined whether there were any differences in reported effects of tryptophan on glucose metabolism between lean and obese mice. The mice fed with high fat diet for 14-weeks (DIO mice) showed significantly higher body weights, fasting blood glucose and serum insulin levels than mice fed with standard diet (control mice) (Fig 3A–3C). In both DIO and control mice, tryptophan ingestion significantly improved glucose tolerance (Fig 3D, 3E, 3G and 3H). However, the extent of improvement was higher in DIO mice (Fig 3D, 3E, 3G and 3H). Greater improvement of glucose tolerance seemed to be caused by the greater enhancement of insulin secretion in DIO mice; 1.81 vs 2.71 folds enhancements, respectively (Fig 3F and 3I). Plasma GIP and GLP-1 levels after glucose load were not significantly affected by tryptophan administration either in DIO or control mice (Fig 3J and 3K). Although GIP levels after tryptophan administration were apparently increased, no statistical differences were observed. As for ghrelin, glucose ingestion suppressed plasma ghrelin levels in DIO (P<0.01) and control mice (with no statistical significance, P = 0.067), tryptophan did not show any effects on the levels (Fig 3L). Glucose ingestion significantly suppressed plasma desacyl ghrelin levels in control mice, and tryptophan attenuated the suppression (Fig 3M). In DIO mice, glucose ingestion with or without tryptophan showed no effects on plasma desacyl ghrelin levels (Fig 3M).

Ob/ob mice at 11 weeks of age showed significantly higher body weights, fasting blood glucose and serum insulin levels than their control littermates (Fig 4A–4C). Similarly, tryptophan significantly improved glucose metabolism in ob/ob mice, while no improvements were observed in control lean mice in the current experimental conditions (Fig 4A, 4B, 4D and 4E). Glucose-stimulated insulin secretion was significantly enhanced by tryptophan administration in ob/ob mice. In control mice, only tendency of enhancement was observed (Fig 4F and 4I). Plasma GIP levels after glucose ingestion were not affected by tryptophan in either ob/ob or control mice (Fig 4J). Plasma GLP-1 levels were significantly elevated only in tryptophan-treated ob/ob mice (Fig 4K), although there were no statistically significant differences in plasma GLP-1 levels at 15 min between vehicle and tryptophan-treated ob/ob mice.

Regarding plasma ghrelin levels, glucose did not suppress ghrelin levels in either ob/ob or control mice with this experimental condition (Fig 4L). Tryptophan significantly elevated plasma ghrelin levels in both ob/ob and control mice (Fig 4L). It also significantly elevated plasma desacyl ghrelin levels in ob/ob mice (Fig 4M).

**Fig 5:**
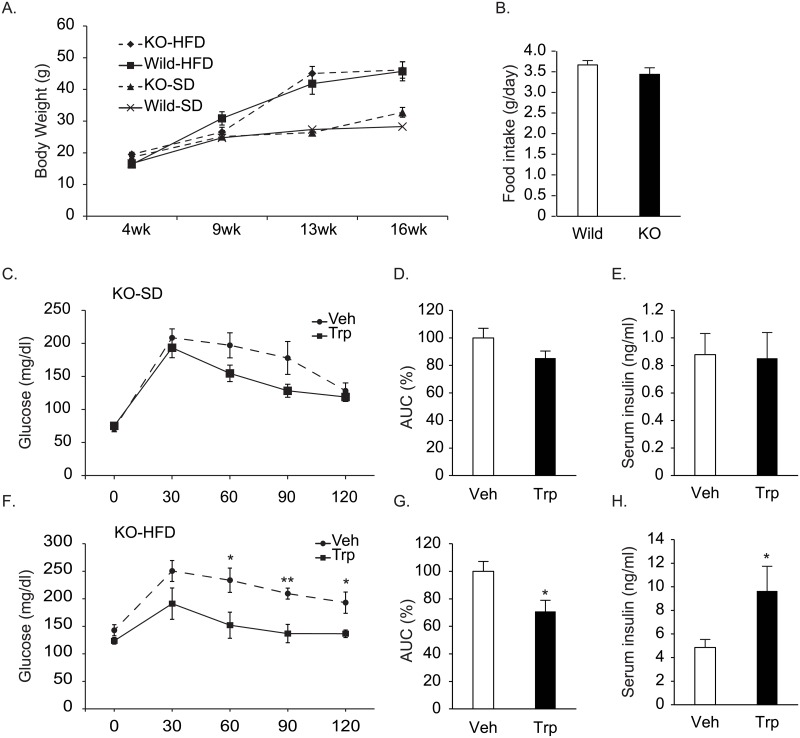
Effects of tryptophan on glucose tolerance in GPR142KO mice. A. Body weight changes of GPR142KO and their wild type littermate mice on standard (SD) and 60% high fat diet (HFD). B. Food intake of 12 weeks-old GPR142KO mice and their wild type littermates. C. Blood glucose levels after oral glucose load (1.0 g/kg) with tryptophan (150mM, 0.5g/kg, Trp) or vehicle (Veh) in GPR142KO (24–26 weeks old) fed with standard diet (C) or high fat diet (F). D, G. Area under the curve (AUC) of glucose levels in C and F. E, H. Serum insulin (E, H) concentrations 15 minutes after glucose load. n = 6–7 per group, *: p<0.05.

We examined the effects of tryptophan on glucose metabolism in GPR142 KO mice to reveal if the tryptophan-induced improvement of glucose tolerance was solely attributed to GPR142-mediated signals (Fig 5). GPR142KO mice showed similar body weights and food intake compared to the wild type littermates (Fig 5A and 5B). They got weights similarly to the control mice when on HFD (Fig 5A). Tryptophan did not improve glucose tolerance in GPR142KO mice significantly (Fig 5C and 5D). Insulin secretion was not stimulated by tryptophan in GPR142KO mice (Fig 5E). In GPR142KO fed with HFD, however, tryptophan significantly improved glucose tolerance with enhanced insulin secretion (Fig 5F–5H), suggesting the involvement of other pathways.

**Fig 6:**
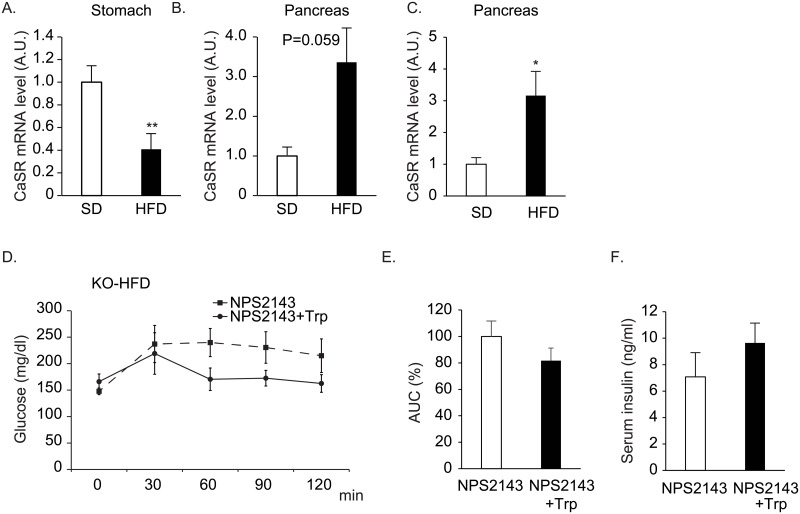
Role of CaSR signaling on the effects of tryptophan in GPR142KO mice. A, B. CaSR mRNA expression levels determined by quantitative RT-PCR in (A) the stomach and pancreas (B) of C57/BL6 mice fed with standard diet (SD) or high fat diet (HFD). n = 6, **: p<0.01.C. CaSR mRNA expression levels in pancreas of GPR142KO mice fed with standard diet (SD) or high fat diet (HFD). n = 6, *: p<0.05. D. Glucose levels after oral glucose load in GPR142KO (22–24 weeks old) mice fed with HFD with L-tryptophan (150 mM, 0.5g/kg, Trp) and NPS 2143(1μM) or NPS2143 alone. E. Area under the curve (AUC) of glucose levels in D. F. Serum insulin concentrations 15 minutes after glucose load. n = 8–9 per group, *: p<0.05.

CaSR has been suggested to be activated by aromatic amino acids including tryptophan and phenylalanine. The expression levels of CaSR mRNA were significantly decreased in the stomach of DIO mice (Fig 6A). The expression levels of CaSR mRNA were increased in the pancreas of wild type (although not statistically significant, P = 0.059; Fig 6B) and GPR142KO mice fed with HFD (P<0.05; Fig 6C). With co-administration of CaSR antagonist, NPS2143, tryptophan did not significantly improve glucose tolerance nor enhanced insulin secretion in GPR142KO mice fed with HFD (Fig 6D–6F).

## Discussion

In this study, we confirmed high expression levels of GPR142 mRNA in the mouse gastrointestinal tracts and pancreatic islets [12]. Previous data indicated GPR142 is highly expressed at least in the ghrelin-producing cells in the stomach and beta cells in the islets [8]. Other reports also indicated that GPR142 expression in the K cells and L cells in the intestine and beta and non-beta cells in the islets [12, 31]. Our findings were consistent with these reports.

We found that GPR142 expression levels in the stomach were increased by fasting and decreased by re-feeding. This regulation is at least partly explained by tryptophan-mediated feed-back down regulation, since direct ingestion of tryptophan downregulated the levels. Regarding chronic regulation, GPR142 mRNA levels in the stomach were suppressed in DIO mice but not in ob/ob mice. Levels were elevated in the pancreas of DIO and ob/ob mice. We also examined the effects of meal-related factors including tryptophan, insulin, glucose, and palmitate on the expression levels of GPR142 in ghrelin-producing cell line MGN3-1 cells and pancreatic beta cell line MIN6 cells (S1 Fig). In MGN3-1 cells, low glucose significantly increased the GPR142 expression level, which may partly explain the elevation of GPR142 by fasting. Tryptophan did not affect GPR142 expression levels, and insulin rather upregulated the level. These results were not consistent with in vivo observation. As for MIN6 cells, no effects were observed by these substances. Further studies will be needed to reveal the mechanism of GPR142 regulation in these organs.

Tryptophan significantly improved glucose tolerance by enhancing insulin secretion as previously reported [12]. Furthermore, we found that the effects were more augmented in DIO mice with enhanced stimulation of GSIS. Notably, we did not observe improved glucose tolerance in littermates control of ob/ob mice. We were not sure for the reason. It may be related to the relatively lighter body weights of littermates control of ob/ob mice when compared to those of control for DIO mice. Previous reports indicated that tryptophan stimulated GIP secretion [12]. In our experimental setting, however, only the tendency of stimulation was observed in both DIO and control mice [12]. GLP-1 levels were also not affected by tryptophan. Therefore, increased tryptophan-induced enhancement of insulin in DIO mice seems to be mostly attributed to the enhanced responses of pancreatic beta cells. The role of incretin seems to be limited.

Although the elevated GPR142 levels in the pancreas of DIO mice may partly contribute to the enhanced tryptophan-induced insulin secretion, it cannot be solely explained by the GPR142 levels, as we still observed augmented tryptophan-induced improvement of glucose tolerance in GPR142KO mice fed with HFD.

CaSR levels were higher in the pancreas of DIO GPR142KO mice, and CaSR antagonist partially blocked the tryptophan-mediated improvement of glucose tolerance and enhancement of GSIS in DIO GPR142KO mice, suggesting that CaSR also contribute to the enhanced GSIS in DIO mice. It should be noted that GPR142KO mice fed with SD showed no statistical significant improvement of glucose tolerance and enhancement of GSIS by tryptophan. GPR142 signaling, therefore, likely has a primary role and CaSR has a limited role in tryptophan-sensing in normal mouse pancreas. CaSR blockade showed effects only in DIO mice. As CaSR antagonist did not completely block the tryptophan’s effects in DIO GPR142KO mice, we could not completely rule out the possibility that other signaling pathways were involved in tryptophan-mediated improvement of glucose tolerance and GSIS enhancement.

Several agonists for GPR142 have been developed by pharmaceutical companies with hope for use as anti-diabetic drugs, as these agonists stimulate glucose-induced insulin secretion in mice [9, 12, 32–37]. These agonists are more effective in obese patients considering the elevated GPR142 expression levels in the pancreas of obese mice, according to our data. Our GPR142KO data suggested, however, that the augmented insulin responses to tryptophan in DIO mice seemed to be also mediated by other pathways including CaSR. Further studies are needed to reveal the effectiveness of these agonists in diabetic patients in various clinical settings.

In conclusion, although GPR142 signaling had a major role in tryptophan recognition for the enhancement of GSIS in lean mice, other pathways including CaSR signaling also had a significant role in obese mice, which seemed to contribute to the augmented enhancement of GSIS by tryptophan in these animals.

## Supporting information

S1 Fig Effects of tryptophan, insulin, glucose, and palmitate on GPR142 expression levels in MGN3-1 cells and MIN6 cells. Effects of 10 mM tryptophan (A, E), 10 μM insulin (B, F), low (100 mg/dl) and high (450 mg/dl) glucose (C, G) and 100 μM palmitate (D, H) on GPR142 expression levels in MGN3-1 and MIN6 cells after 2 hours incubation. n = 6, *: p<0.05, **: p<0.01.
(EPS)
